# Lower systemic nitric oxide bioactivity, cerebral hypoperfusion and accelerated cognitive decline in formerly concussed retired rugby union players

**DOI:** 10.1113/EP091195

**Published:** 2023-07-09

**Authors:** Thomas S. Owens, Christopher J. Marley, Thomas A. Calverley, Benjamin S. Stacey, Lewis Fall, Hayato Tsukamoto, Angelo Iannetelli, Teresa Filipponi, Bruce Davies, Gareth L. Jones, Christophe Hirtz, Sylvain Lehmann, Edouard Tuaillon, Nicola Marchi, Damian M. Bailey

**Affiliations:** ^1^ Neurovascular Research Laboratory, Faculty of Life Sciences and Education University of South Wales UK; ^2^ Faculty of Computing, Engineering and Science University of South Wales UK; ^3^ Faculty of Sport Sciences Waseda University Tokorozawa, Japan; ^4^ LBPC‐PPC University of Montpellier, Institute of Regenerative Medicine‐Biotherapy IRMB, Centre Hospitalier Universitaire de Montpellier, INSERM Montpellier France; ^5^ CHU Montpellier, Department of Bacteriology‐Virology Centre University of Montpellier France; ^6^ Cerebrovascular and Glia Research, Department of Neuroscience Institute of Functional Genomics (University of Montpellier, CNRS, INSERM) Montpellier France

**Keywords:** cerebrovascular function, concussion, nitric oxide, rugby union

## Abstract

Following retirement from sport, the chronic consequences of prior‐recurrent contact are evident and retired rugby union players may be especially prone to accelerated cognitive decline. The present study sought to integrate molecular, cerebrovascular and cognitive biomarkers in retired rugby players with concussion history. Twenty retired rugby players aged 64 ± 5 years with three (interquartile range (IQR), 3) concussions incurred over 22 (IQR, 6) years were compared to 21 sex‐, age‐, cardiorespiratory fitness‐ and education‐matched controls with no prior concussion history. Concussion symptoms and severity were assessed using the Sport Concussion Assessment Tool. Plasma/serum nitric oxide (NO) metabolites (reductive ozone‐based chemiluminescence), neuron specific enolase, glial fibrillary acidic protein and neurofilament light‐chain (ELISA and single molecule array) were assessed. Middle cerebral artery blood velocity (MCAv, doppler ultrasound) and reactivity to hyper/hypocapnia (${\mathrm{CVR}}_{{\mathrm{CO}}_{2}\mathrm{hyper}}$/${\mathrm{CVR}}_{{\mathrm{CO}}_{2}\mathrm{hypo}}$) were assessed. Cognition was determined using the Grooved Pegboard Test and Montreal Cognitive Assessment. Players exhibited persistent neurological symptoms of concussion (*U* = 109_(41)_, *P* = 0.007), with increased severity compared to controls (*U* = 77_(41)_, *P* < 0.001). Lower total NO bioactivity (*U* = 135_(41)_, *P* = 0.049) and lower basal MCAv were apparent in players (*F*_2,39_ = 9.344, *P* = 0.004). This was accompanied by mild cognitive impairment (*P* = 0.020, 95% CI, −3.95 to −0.34), including impaired fine‐motor coordination (*U* = 141_(41)_, *P* = 0.021). Retired rugby union players with history of multiple concussions may be characterised by impaired molecular, cerebral haemodynamic and cognitive function compared to non‐concussed, non‐contact controls.

## Abstract (highlights)

- **What is the central question of this study?**
  What are the molecular, cerebrovascular and cognitive biomarkers of retired rugby union players with concussion history?
- **What is the main finding and its importance?**
  Retired rugby players compared with matched controls exhibited lower systemic nitric oxide bioavailability accompanied by lower middle cerebral artery velocity and mild cognitive impairment. Retired rugby players are more susceptible to accelerated cognitive decline.

## 1 INTRODUCTION

The chronic consequences of prior recurrent contact and concussion are most evident following retirment from sport. Fuller et al. (2007) identified that rugby union players may be exposed to as many as 11,000 contact events per‐season, equating to nearly a quarter of a million contact events over a 20‐year playing career. Compared to non‐contact controls, retired athletes with three or more concussions are characterised by a fivefold risk of mild cognitive impairment (MCI) and are more susceptible to neurodegeneration, including chronic traumatic encephalopathy (CTE; Gardner, Iverson, McCrory, 2014; Guskiewicz et al., 2005). This extends to other contact sports including American Football, soccer and ice hockey (Gardner, Iverson, McCrory, 2014). While rugby union has one of the highest concussion incidence rates of any sport (England Rugby, 2020; Gardner, Iverson, Williams et al., 2014), the confirmed diagnosis of CTE among retired rugby players is sparse. Stewart et al. (2016) attributed this to a limited number of brain donations, whereas Lee et al. (2019) suggested that mixed neurodegenerative pathology during autopsy may lead to misdiagnosis.

Retired rugby union players may be uniquely susceptible to accelerated cognitive impairment due to repetitive exposure to contact and concussion. While the precise underlying mechanisms remain to be established, several have been proposed. Cerebral hypoperfusion has been consistently associated with MCI and neurodegeneration (Johnson et al., 2005; Wolters et al., 2017) and is attributable to two potential mechanisms. First, the neurometabolic cascade of concussion promotes mitochondrial dysfunction and an elevation in free radicals that have the thermodynamic potential to scavenge nitric oxide (NO), collectively referred to as oxidative‐nitrosative stress (OXNOS), which can impair cerebrovascular function (Bailey et al., 2019; Bailey, Marley et al., 2013; Bailey, Rimoldi et al., 2013; Giza & Hovda, 2014; Owens et al., 2021). Second, shear stress imposed by mechanical forces associated with contact can lead to the structural loss of neuronal network integrity and promote atrophy (Grossman et al., 2013). These events may be further compounded by ageing and physical inactivity, as biological ageing is associated with up to a 30% reduction in basal cerebral perfusion and vasoreactivity across the adult lifespan (Ainslie et al., 2008; Bailey, Marley et al., 2013)

At present, no studies have examined the functionally integrated mechanisms underlying cognitive decline in retired rugby union players. To address this, we examined molecular, cerebrovascular and cognitive biomarkers in retired rugby players with an established history of concussion specifically caused by recurrent contact. We hypothesised that compared to non‐contact, apparently healthy matched controls, players would exhibit a lower systemic concentration of bioactive NO that would be accompanied by impaired cerebrovascular and cognitive function.

## 2 METHODS

### 2.1 Ethical approval

The study was approved by the University of South Wales ethics committee (no. 2017TO1102). Verbal and written informed consent were obtained from all participants and anonymity ensured via a random code generator. All experimental procedures conformed to the standards set by the *Declaration of Helsinki*, except for registration in a database (Williams, 2008).

### 2.2 Design

A cross‐sectional observational study was conducted. Following confirmation of interest, potential participants were contacted via telephone and completed an eligibility questionnaire during screening. If eligible, participants were invited to attend the Neurovascular Research Laboratory (University of South Wales, UK) on two separate occasions.

#### 2.2.1 Participants

Forty‐four males were divided into two subgroups. Twenty‐two retired rugby players aged 64 ± 5 years reporting three (interquartile range (IQR), 3; min = 1, max = 10) concussions incurred over 22 (IQR, 6) years of play at regional and international level were compared to 22 age‐, cardiorespiratory fitness (CRF)‐ and education‐matched controls with no participation in contact sports or concussion history (Table 1). Players were included in the study upon confirmation of at least one self‐reported or medically diagnosed concussion. All participants were asked to undergo dietary control by maintaining their ‘normal’ dietary behaviours for the duration of the study. Participants were further instructed to refrain from physical activity, caffeine and alcohol, follow a low nitrate/nitrite diet and complete a 12 h overnight fast prior to both experimental visits (Bailey et al., 2017; Wang et al., 1997). No participants were taking any over‐the‐counter antioxidant supplements. As anticipated in this (aged) demographic, several participants were prescribed medication for the specific treatment of hypertension, hypertriglyceridaemia and coagulopathies (Table 1).

**TABLE 1:** Demographics.

	Controls (*n* = 21)	Players (*n* = 20)	*P*	95% CI
Anthropometrics				
Stature (cm)	174 ± 7	176 ± 9	0.324	−2.50 to 7.37
Mass (kg)	84 ± 20	89 ± 14	0.327	−5.68 to 16.61
BMI (kg/m^2^)	28 ± 5	29 ± 4	0.395	−1.68 to 4.16
Body fat (%)	24 ± 9	24 ± 6	0.989	−4.95 to 5.03
Clinical				
Age (years)	64 ± 7	64 ± 5	0.992	−3.73 to 3.77
Total education (years)	16 ± 3	17 ± 3	0.303	−0.87 to 2.74
Alcohol consumption (units/week)	8 (8)	14 (11)	0.381	—
Systolic blood pressure (mmHg)	147 ± 15	148 ± 17	0.186	−1.53 to 7.63
Diastolic blood pressure (mmHg)	84 ± 8	87 ± 6	0.424	−3.46 to 8.05
Mean arterial pressure (mmHg)	105 ± 10	107 ± 9	0.904	−5.05 to 5.70
${\dot{V}}_{O_{2}\max}$ (ml/kg/min)	24 (14)	28 (10)	0.927	—
Contact/activity				
Concussions (*n*)	0 (0)	3 (3)	**0.000**	—
Rugby career (years)	0 (0)	22 (6)	**0.000**	—
Total physical activity (min/week)	377 ± 293	380 ± 132	0.565	−142.40 to 147.12
Resistance activity (min/week)	30 (120)	90 (128)	0.090	—
Aerobic activity (min/week)	360 (308)	270 (105)	0.917	—
Medication				LH ratio
Aspirin (*n* (%))	1 (5)	2 (10)	0.517	0.421
ACE inhibitors (*n* (%))	3 (14)	4 (20)	0.674	0.177
Angiotensin receptor blockers (*n* (%))	1 (5)	0 (0)	0.517	0.421
Calcium channel antagonists (*n* (%))	2 (10)	2 (10)	0.674	0.177
Clopidogrel (*n* (%))	0 (0)	1 (5)	0.227	1.462
Diuretics (*n* (%))	1 (5)	1 (5)	0.972	0.001
Statins (*n* (%))	6 (29)	4 (20)	0.939	0.006
Warfarin (*n* (%))	0 (0)	1 (5)	0.227	1.462

Values are means ± SD or medians (interquartile range), medication presented as frequency and percentage of cohort. Values in bold indicate *P* < 0.05*vs*. controls.

Abbreviations: ACE, angiotensin‐converting enzyme; BMI, body mass index; LH, likelihood; ${\dot{V}}_{O_{2}\max}$, maximal oxygen uptake.

### 2.3 Experimental procedures: visit one

#### 2.3.1 Medical examination

Participants completed a comprehensive medical screen and 12‐lead functional diagnostic exercise test (below). The attending clinician confirmed information provided by the participant during the telephone screening. Following confirmation, medication (Table 1), family history of any major diseases and/or conditions were recorded. Participants were asked to confirm any chest pain at rest, in response to, or following exercise in the 3 months preceding experimental visits, including any indications of dyspnoea, syncope, palpitations or cerebrovascular symptoms aligned to transient ischaemic episodes, or peripheral vascular symptoms indicative of claudication. In those participants prescribed medication, a review was conducted with regard to any recent changes in dosage and/or formulation. A brief physical examination was then conducted. Blood pressure (BP, auscultation) and heart rate (HR) were recorded after 10 min of supine rest. Participants were screened clinically for irregular pulse, presence of a murmur or additional heart sounds using a stethoscope (Littmann Classic III, 3M, Bracknell, UK). Brief respiratory observations were completed for the nose and throat, followed by percussion and auscultation of the chest (Bailey et al., 2000).

#### 2.3.2 Dietary assessment

Given the link between dietary intake and systemic OXNOS reflected by a free radical‐mediated reduction in vascular NO bioavailability (Bailey, Culcasi et al., 2022), dietary intake was assessed. This was completed using a self‐administered validated semi‐quantitative food frequency questionnaire (FFQ) aimed to estimate a participant's typical food intake over the past 12 months (Bingham et al., 1997; McKeown et al., 2001). The FFQ included quantitative questions on frequency of consumption of generic food groups (i.e., cereals, meats, fish and dairy products, fats and oils, sweet foods and snacks, fruits, vegetables, and drinks). Participants were asked to indicate their usual rate of consumption for each item, ranging from ‘rarely or never’ to ‘6+ times per week’. Dietary data were converted into estimated nutrient intakes using the dietary software package Q‐Builder (Tinuviel Software, Anglesey, UK).

#### 2.3.3 Electrocardiography

Participants were fitted with a 12‐lead ECG (Welch Allyn, Aylesbury, UK) to exclude myocardial abnormalities. Participants remained at rest in a supine position for 10 min. Thereafter, a continuous recording of the ECG was performed both at rest and over the course of a standardised exercise stress test (below).

#### 2.3.4 Exercise stress test

Participants were seated on an electronically braked semi‐recumbent cycle ergometer (Lode Corival, Cranlea & Company, Birmingham, UK) and completed an incremental exercise test to exhaustion. Online breath‐by‐breath respiratory gas analysis was performed throughout (Ultima Series, Medgraphics, King's Lynn, UK). HR was monitored throughout exercise using the 12‐lead ECG. Following a 2‐min rest period, participants were instructed to pedal at 70 revolutions per minute (r.p.m.) and completed a 2‐min warm‐up period under no ergometer load. Ergometer workload was calculated via established criteria ensuring automated gradual increases to exercise intensity based on an age and mass predictive algorithm until volitional exhaustion (Wasserman, 2012). Peak exercise performance was confirmed if participants achieved three of the following (five) criteria: (i) participant oxygen consumption (${\dot{V}}_{O_{2}}$) remained unchanged under increasing ergometer load, (ii) cadence fell below 60 r.p.m., (iii) respiratory exchange ratio (RER) in excess of 1.15 arbitrary units (AU), (iv) HR within 10% of age predicted maximum, and (v) rating of perceived exertion of 20 points (Rose et al., 2018). Participants completed a 2‐min cool‐down (unloaded) and a further 10 min of supine recovery.

### 2.4 Experimental procedures: visit two

#### 2.4.1 Clinical function: cognition

The Montreal Cognitive Assessment (MoCA) was used to assess the following cognitive domains: attention and concentration, executive function, memory, language, visuoconstructional skills, conceptual thinking, calculations and orientation; and to screen for MCI. A score below 26 on the MoCA was used to determine MCI in accordance with established recommendations (Nasreddine et al., 2005). Fine motor processing and coordination were assessed via the Grooved Pegboard Dexterity test using the dominant (GPD) and non‐dominant (GPND) hands (Trites, 1977). Superior performance was indicated by higher scores in the MoCA, or lower scores in GPD and GPND (Marley et al., 2017).

#### 2.4.2 Sports concussion assessment

Participants completed the off‐field section of the Sports Concussion Assessment Tool (SCAT5, McCrory et al., 2017). Cognitive screening, neurological screening and delayed recall were performed according to established recommendations (Davis et al., 2017). A symptom evaluation was used to assess prolonged symptomology and severity. Players self‐recalled an average of 32 ± 12 years since their most recent concussion and returned to play granted following 8 ± 14 days. Controls reported no concussion history.

#### 2.4.3 Molecular function

Blood was collected into vacutainers from an indwelling cannula located in a forearm antecubital vein (BD Biosciences, Wokingham, UK) and centrifuged at 600 *g* (4°C) for 10 min. Plasma supernatant was decanted into cryogenic vials (Nalgene Labware, Thermo Fisher Scientific Inc., Waltham, MA, USA) and snap‐frozen in liquid nitrogen. Samples were thawed at 37°C prior to batch analysis.

##### Nitric oxide metabolites

Reductive ozone‐based chemiluminescence (Sievers NOA 280i, Analytix Ltd, Durham, UK) was used to assess plasma NO bioavailability, determined as the cumulative concentration of nitrite (NO_2_^−^) and *S*‐nitrosothiols (RSNO) as previously described (Bailey et al., 2017). Intra‐ and inter‐assay coefficients of variation (CVs) were both <5%.

##### Neurovascular unit integrity/injury

A panel of serum biomarkers were quantified using ultrasensitive analytical platforms in a subset of 10 randomly selected retired players and 10 controls as recently described (Bailey, Bain et al., 2022). Glial fibrillary acidic protein (GFAP), an intermediate filament protein expressed predominantly by astrocytes (Bignami et al., 1972), was employed as a biomarker of blood–brain barrier (BBB) permeability and gliovascular damage. Neuron specific enolase (NSE), an intracytoplasmic glycolytic enzyme derived from the neuronal cytoplasm and neuroendocrine cells (Pahlman et al., 1984), and neurofilament light‐chain (NFL), a component of the axonal cytoskeleton expressed primarily in large‐calibre myelinated subcortical axons (Friede & Samorajski, 1970), reflect neuronal–axonal damage.

The underlying source(s), biochemistry, detection and clinical interpretation of these biomarkers have recently been reviewed (Janigro et al., 2021). Determination of NSE was performed using a fully automated CE‐IVD chemiluminescence immunoassay (Liaison, DiaSorin S.p.A, Saluggia, Italy). We employed the Neurology 4‐Plex assay kit (Quanterix Corp., Lexington, MA, USA) (Rissin et al., 2010) to measure GFAP and NFL proteins on a single molecule array (Simoa) HD‐1 Analyzer (Quanterix Corp). Based on singulation of enzyme labelled immune‐complex on paramagnetic beads, this digital ELISA assay is considered ∼1200‐fold more sensitive than conventional immunoassays (Wilson et al., 2016). All samples were analysed following (4‐fold) dilution with the diluent provided in the kit (phosphate buffer with bovine serum and heterophilic blocker solution) to minimise matrix effects. The intra‐ and inter‐assay CVs for all metabolites were <5%.

#### 2.4.4 Cerebral haemodynamic function

##### Perfusion

Middle cerebral artery (MCA) blood velocity (MCAv; Skow et al., 2022) was determined using a 2 MHz pulsed Doppler ultrasound system (TCD, Multi‐Dop X4, DWL Elektroniche Systeme GmbH, Sipplingen, Germany). Insonation of the M1 segment of the right (or left, when insonation of the right MCA side was unachievable) MCA was conducted at depths of 45–60 mm (Aaslid et al., 1989). The probe was secured over the trans‐temporal window using a headband in order to attain optimal insonation and prevent movement artefact. Beat‐by‐beat mean arterial blood pressure (MAP), HR and cardiac output ($\dot{Q}$) were recorded continuously using finger photoplethysmography (Finometer PRO; Finapres Medical Systems, Amsterdam, The Netherlands). Cerebrovascular and (systemic) total peripheral resistance (CVRi and TPR) were calculated as MAP/MCAv or $\dot{Q}$, respectively. Cerebrovascular conductance index (CVCi) was calculated as MCAv/MAP and pulsatility index (PI) calculated as systolic MCAv (SMCAv) − diastolic MCAv (DMCAv)/MCAv.

Cerebral oxygen delivery (${\mathrm{CD}}_{O_{2}}$) was calculated as MCAv × arterial O_2_ content ($C_{{\mathrm{aO}}_{2}}$) (1.39 × Hb × $S_{{\mathrm{aO}}_{2}}$/100) ignoring the negligible concentration of dissolved O_2_. Since we did not perform arterial blood gas analysis, $S_{{\mathrm{aO}}_{2}}$ was determined via fingertip pulse oximetry (Nonin Onyx II 9550, Nonin, Plymouth, MN, USA (Basaranoglu et al., 2015) and haemoglobin (Hb) assayed photometrically (HemoCue, Kuvettgatan, Sweden) to complete the derivation (Bailey et al., 2019).

##### Reactivity

Cerebrovascular reactivity to hypercapnia (${\mathrm{CVR}}_{{\mathrm{CO}}_{2}\mathrm{hyper}}$; 5% CO_2_, balanced air) and hypocapnia (${\mathrm{CVR}}_{{\mathrm{CO}}_{2}\mathrm{hypo}}$; hyperventilation at 15 breaths/min) were assessed for 3 min and calculated as the percentage change in MCAv from baseline per mmHg change in end‐tidal CO_2_ ($P_{{\mathrm{ETCO}}_{2}}$) determined via capnography (ML 206, ADInstruments Ltd, Oxford, UK). The fractional sums of ${\mathrm{CVR}}_{{\mathrm{CO}}_{2}\mathrm{hyper}}$ and ${\mathrm{CVR}}_{{\mathrm{CO}}_{2}\mathrm{hypo}}$ were used to calculate CVR range (${\mathrm{CVR}}_{{\mathrm{CO}}_{2}\mathrm{range}}$) as previously described (Bailey, Jones et al., 2013). The absolute change (Δ) in MCAv was also determined during from rest to hyper/hypocapnia. All data were sampled continuously at 1 kHz (Powerlab, ADInstruments, Colorado Springs, CO, USA) and stored for off‐line analysis.

### 2.5 Statistical analysis

Data were analysed using a commercially available statistical package (SPSS Statistics 29.0, IBM Corp., Armonk, NY, USA). Following confirmation of distribution normality with the Shapiro–Wilk *W*‐test (*P* > 0.05), between‐group differences were determined with Student's independent samples *t*‐test for participant demographics, dietary intake, molecular parameters, cognition, CVR and SCAT5 datasets. A two‐way Group (controls *vs*. players) × Condition (normocapnia *vs*. hyper/hypocapnia) analysis of variance (ANOVA) was conducted to determine differences between groups for changes in cerebrovascular function. Following an interaction, *post hoc* analyses were performed using Bonferonni‐corrected paired and independent sample *t*‐tests. Associations for the expected and observed number of participants who were prescribed medication or experiencing persistent concussion symptoms between groups were determined using likelihood ratios (LH; Özdemir & Eyduran, 2005). Relationships between select molecular, cerebral haemodynamic and cognitive metrics were assessed using Pearson's product moment correlation. Results are expressed as the mean ± standard deviation (SD) or median (IQR) if not normally distributed. Significance for all two‐tailed tests was established at *P* < 0.05.

## 3 RESULTS

Three participants were excluded from the study due to ill‐health (*n* = 2) and failure to comply with the complete experimental protocol (*n* = 1). Forty‐one participants were included in the final analysis: 20 retired players and 21 matched controls.

### 3.1 Demographics and dietary intake

By design, both groups were matched for age, anthropometrics, physical activity, CRF, education level and medication (*P* > 0.05, Table 1). However, retrospective analyses (i.e. beyond our immediate control) revealed that players consumed less calories (*P* = 0.035, 95% CI, −708.16 to −27.73), carbohydrate (*U* = 114_(41)_, *P* = 0.012), protein (*P* = 0.043, 95% CI, −28.87 to −0.49), chloride (*U* = 120_(41)_, *P* = 0.019), copper (*P* = 0.002, 95% CI, −0.62 to −0.15), sodium (*U* = 118_(41)_, *P* = 0.016), iron (*P* = 0.008, 95% CI, −4.06 to −0.64), manganese (*P* = 0.009, 95% CI, −1.72 to −0.27), selenium (*P* = 0.012, 95% CI, −23.04 to −3.06), folate (*P* = 0.040, 95% CI, −146.34 to −29.58) and thiamine (*P* = 0.003, 95% CI, −0.063 to −0.14) compared to controls (Table 2).

**TABLE 2:** Dietary intake.

	Controls (*n* = 21)	Players (*n* = 20)	*P*	95% CI	Guidelines UK^a^
Calories (kcal)	2316 ± 529	1948 ± 547	**0.035**	−708.16 to −27.73	2581
Carbohydrate (g)	284 (65)	216 (81)	**0.012**	—	—
Fat (g)	90 ± 21	76 ± 29	0.078	−30.27 to 1.50	—
Protein (g)	97 ± 22	83 ± 23	**0.043**	−28.87 to −0.49	—
Free sugars (g)	56 (13)	34 (21)	**0.004**	—	—
Vitamin E (mg)	9.20 ± 2.56	8.95 ± 3.91	0.805	−2.33 to 1.82	>4 mg/d^b^
Vitamin C (mg)	180 (62)	133 (69)	0.083	–	40 mg/d^c^
Folate (μg)	415 ± 103	327 ± 81	**0.04**	−146.34 to −29.58	200 μg/d^c^
Thiamine (mg)	1.9 ± 0.4	1.5 ± 0.3	**0.003**	−0.63 to −0.14	0.9 mg/d^c^
Chloride (mg)	5050 (1244)	3992 (1548)	**0.019**	—	2500 mg/d^c^
Copper (mg)	1.47 ± 0.41	1.08 ± 0.33	**0.002**	−0.62 to −0.15	1.2 mg/d^c^
Sodium (mg)	3216 (748)	2475 (791)	**0.016**	–	1600 mg/d^c^
Iron (mg)	13 ± 3	11 ± 3	**0.008**	−4.06 to −0.64	8.7 mg/d^c^
Manganese (mg)	4.31 ± 1.19	3.32 ± 1.11	**0.009**	−1.72 to −0.27	>1.4 mg/d^b^
Selenium (μg)	58 ± 17	45 ± 14	**0.012**	−23.04 to −3.06	75 μg/d^c^

Values are means ± SD or medians (interquartile range).

^a^ Department of Health UK Guidelines.

^b^ Safe intake.

^c^ Reference nutrient intake. Values in bold indicate *P* < 0.05*vs*. controls. Abbreviation: BMI, body mass index.

### 3.2 Concussion symptoms

Players exhibited persistent neurological symptoms of concussion (*U* = 109_(41)_, *P* = 0.007; Table 3) and the severity was elevated compared to controls (*U* = 77_(41)_, *P* < 0.001). Players were more likely to report consistent symptoms of headache (LH = 5.002, *P* = 0.025), pressure in head (LH = 7.912, *P* = 0.005), neck pain (LH = 9.866, *P* = 0.002), blurred vision (LH = 7.912, *P* = 0.005), sensitivity to light (LH = 6.199, *P* = 0.013), ‘don't feel right’ (LH = 5.002, *P* = 0.025), difficulty concentrating (LH = 5.664, *P* = 0.017), difficulty remembering (LH = 14.191, *P* < 0.001), ‘more emotional’ (LH = 9.502, *P* = 0.009), nervous or anxious (LH = 4.822, *P* = 0.028) and trouble falling asleep (LH = 7.376, *P* = 0.025).

**TABLE 3:** Sports concussion assessment.

Parameter	Controls (*n* = 21)	Players (*n* = 20)	*P*	95% CI
Total concussion symptoms (*n*)	2 (5)	6 (6)	**0.007**	—
Concussion severity score (*n*)	0 (5)	12 (15)	**0.000**	—
Orientation (*n*)	5 (1)	5 (0)	0.138	–
Immediate recall (*n*)	17 ± 3	18 ± 4	0.545	−1.60 to 2.98
Concentration (*n*)	4 (2)	4 (2)	0.359	—
Balance Errors (*n*)	8 ± 5	9 ± 5	0.453	−1.83 to 4.03
Delayed Recall (*n*)	2 (4)	3 (1)	0.382	—
Symptoms (frequency)				LH ratio
Headache	1	6	**0.025**	5.002
Pressure in head	0	5	**0.005**	7.912
Neck pain	1	8	**0.002**	9.866
Nausea or vomiting	0	1	0.227	1.462
Dizziness	1	4	**0.058**	3.603
Blurred vision	0	5	**0.005**	7.912
Balance problems	2	6	0.093	2.829
Sensitivity to light	0	3	**0.013**	6.199
Sensitivity to noise	0	4	**0.005**	7.192
Feeling slowed down	3	6	0.119	2.431
Feeling like ‘in a fog’	0	4	**0.013**	6.199
Don't feel right	1	6	**0.025**	5.002
Difficulty concentrating	5	11	**0.017**	5.664
Difficulty remembering	6	15	**<0.001**	14.191
Fatigue or low energy	5	8	0.151	2.066
Confusion	2	2	0.959	0.003
Drowsiness	4	4	0.645	0.212
More emotional	3	10	**0.009**	9.502
Irritability	4	7	0.072	3.245
Sadness	1	7	**0.004**	8.195
Nervous or anxious	3	8	**0.028**	4.822
Trouble falling asleep	2	7	**0.025**	7.376

Values are means ± SD or medians (interquartile range). *P*‐values in bold indicate *P* < 0.05*vs*. controls. Abbreviation: LH, likelihood.

### 3.3 NO bioavailability

**FIGURE 1:**
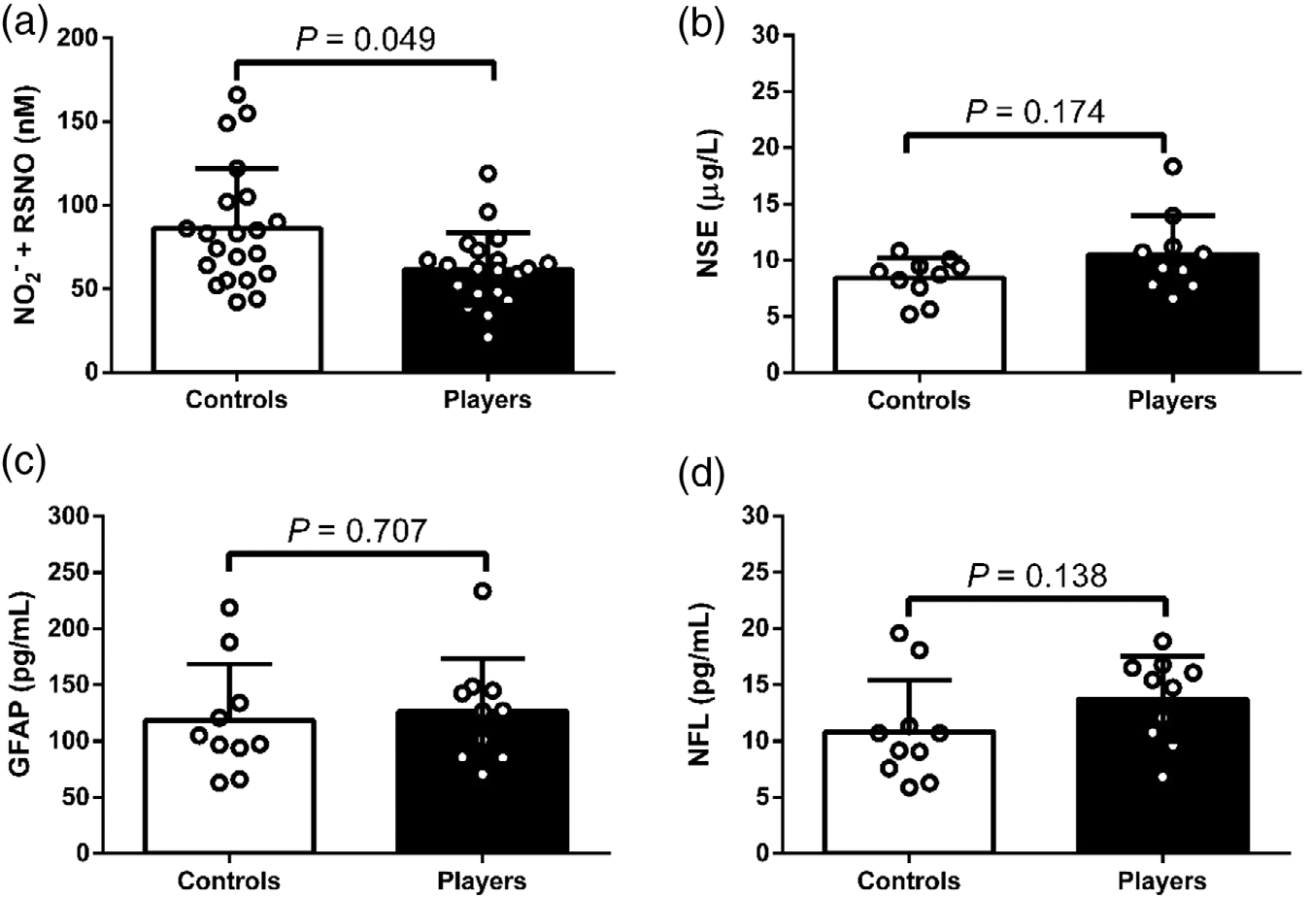
Basal nitric oxide bioactivity (a), neuron specific enolase (b), glial fibrillary acidic protein (c) and neurofilament light‐chain (d). Values are means ± SD. Panel (a): controls, *n* = 21; players, *n* = 20. Panels (b–d): controls, *n* = 10; players, *n* = 10. GFAP, glial fibrillary acidic protein; NFL, neurofilament light‐chain; NO_2_^−^, nitrite; NSE, neuron specific enolase; RSNO, *S*‐nitrosothiols.

Compared to controls, players exhibited lower basal bioactive NO (*U* = 135_(41)_, *P* = 0.049; Figure 1a).

### 3.4 Neurovascular unit integrity

No differences in NSE (*U* = 32_(20)_, *P* = 0.174, β = 0.369), GFAP (*P* = 0.707, 95% CI, −55.83 to 37.28, β = 0.069) or NFL (*P* = 0.138, 95% CI, −6.89 to 1.03, β = 0.313) were observed between players and controls (Figure 1b–d).

### 3.5 Cerebrovascular function

#### 3.5.1 Absolute changes

**FIGURE 2:**
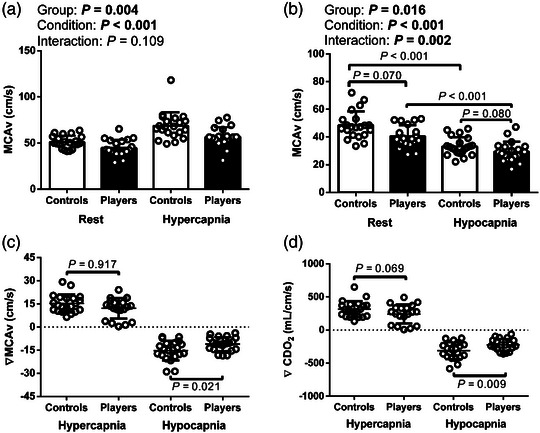
(a, b) Cerebral blood velocity at rest, hypercapnia (a) and hypocapnia (b). (c, d) Change (Δ) in cerebral blood velocity (CBv, c) and cerebral oxygen delivery (${\mathrm{CD}}_{O_{2}}$, d) during the CO_2_ challenges. Values are means ± SD; controls, *n* = 21; players, *n* = 20. MCAv, middle cerebral artery blood velocity.

At rest and during exposure to hypercapnia (Table 4 and Figure 2a), players exhibited lower MCAv (*F*_2,39_ = 9.344, *P* = 0.004), SMCAv (*F*_2,39_ = 11.019, *P* = 0.002), DMCAv (*F*_2,39_ = 5.133, *P* = 0.029) and subsequent ${\mathrm{CD}}_{O_{2}}$ (*F*_2,39_ = 12.353, *P* = 0.001). Players also exhibited higher CVRi (*F*_2,39_ = 4.351, *P =* 0.044) and lower TPR compared to controls (*P* = 0.028, 95% CI, −3.08 to −0.088). During hypocapnia (Table 5), players exhibited lower SMCAv (*P* = 0.020, 95% CI, −14.85 to −1.36) and ${\mathrm{CD}}_{O_{2}}$ relative to controls (*P* = 0.021, 95% CI, −188.29 to −16.53). The change in MCAv (*P* = 0.021, 95% CI, 0.67–7.63; Figure 2c) and ${\mathrm{CD}}_{O_{2}}$ (*P* = 0.009, 95% CI, 24.15–161.71; Figure 2d) in response to hypocapnia was lower in players compared to controls.

**TABLE 4:** Cerebrovascular and cardiovascular responses to hypercapnia.

	Controls (*n* = 21)			Players (*n* = 20)			*P*		
Condition	Baseline	Hypercapnia	Δ	Baseline	Hypercapnia	Δ	Group	Condition	Interaction
Cerebrovascular									
MCAv (cm/s)	51 ± 7	69 ± 15	17 ± 12	45 ± 9	57 ± 10	12 ± 7	**0.004**	**0.000**	0.076
SMCAv (cm/s)	85 ± 10	109 ± 22	24 ± 16	73 ± 14	87 ± 27	14 ± 22	**0.002**	**0.000**	0.104
DMCAv (cm/s)	32 ± 6	44 ± 11	12 ± 7	29 ± 7	37 ± 8	9 ± 5	**0.029**	**0.000**	0.107
CVRi (mmHg/cm/s)	1.87 ± 0.38	1.52 ± 0.33	−0.34 ± 0.20	2.18 ± 0.56	1.82 ± 0.64	−0.36 ± 0.32	**0.044**	**0.000**	0.879
CVCi (cm/s/mmHg)	0.55 ± 0.12	0.67 ± 0.18	0.12 ± 0.10	0.48 ± 0.16	0.67 ± 0.33	0.18 ± 0.23	0.560	**0.000**	0.251
PI (AU)	1.05 ± 0.23	0.98 ± 0.20	−0.08 ± 0.13	1.00 ± 0.16	0.97 ± 0.17	−0.04 ± 0.08	0.631	**0.002**	0.250
CD_O__2_ (ml/cm/s)	1048 ± 170	1400 ± 283	352 ± 212	878 ± 184	1118 ± 242	243 ± 144	**0.001**	**0.000**	0.056
Cardiopulmonary									
HR (bpm)	65 ± 16	63 ± 12	‐3 ± 9	60 ± 9	60 ± 8	1 ± 3	0.278	0.399	0.100
MAP (mmHg)	92 ± 13	102 ± 17	10 ± 13	94 ± 19	99 ± 23	4 ± 9	0.905	**0.000**	0.135
TPR (mmHg/l/min)	15.35 ± 4.19	17.12 ± 4.21^*^	1.77 ± 2.81	16.34 ± 4.15	16.52 ± 4.34	0.19 ± 1.83^†^	0.879	**0.012**	**0.039**
$P_{{\mathrm{ETCO}}_{2}}$ (mmHg)	42 ± 3	53 ± 3	11 ± 4	42 ± 4	52 ± 5	10 ± 2	0.901	**0.000**	0.135

Values are means ± SD. ^†^ *P* < 0.05 *vs*. controls. ^*^ *P* < 0.05 within groups. *P*‐values in bold indicate statistical significance. Abbreviations: ${\mathrm{CD}}_{O_{2}}$, cerebral oxygen delivery; CVCi, cerebrovascular conductance index; CVRi, cerebrovascular resistance index; DMCAv, diastolic middle cerebral artery blood velocity; HR, heart rate; MAP, mean arterial pressure; MCAv, middle cerebral artery blood velocity; $P_{{\mathrm{ETCO}}_{2}}$, end tidal carbon dioxide; PI, pulsatility index; SMCAv, systolic middle cerebral artery blood velocity; TPR, total peripheral resistance.

**TABLE 5:** Cerebrovascular and cardiovascular responses to hypocapnia.

	Controls (*n* = 21)			Players (*n* = 20)			*P*		
Condition	Baseline	Hypocapnia	%Δ	Baseline	Hypocapnia	%Δ	Group	Condition	Interaction
Cerebrovascular									
MCAv (cm/s)	49 ± 10	33 ± 7^*^	−15 ± 6	41 ± 8^†^	29 ± 7^*^	−11 ± 5	**0.016**	**0.000**	**0.022**
SMCAv (cm/s)	81 ± 15	63 ± 10^*^	−18 ± 9	67 ± 12^†^	55 ± 11^†*^	−12 ± 8	**0.004**	**0.000**	**0.022**
DMCAv (cm/s)	30 ± 8	19 ± 5	−11 ± 5	25 ± 6	16 ± 6	−9 ± 5	0.059	**0.000**	0.179
CVRi (mmHg/cm/s)	2.02 ± 0.47	2.69 ± 0.65	0.67 ± 0.37	2.28 ± 0.75	3.03 ± 1.17	0.75 ± 0.84	0.190	**0.000**	0.707
CVCi (cm/s^/^mmHg)	0.51 ± 0.13	0.40 ± 0.09	−0.11 ± 0.08	0.49 ± 0.21	0.38 ± 0.14	−0.12 ± 0.22	0.537	**0.000**	0.882
PI (AU)	1.08 ± 0.25	1.36 ± 0.30	0.28 ± 0.20	1.05 ± 0.17	1.37 ± 0.45	0.32 ± 0.37	0.854	**0.000**	0.648
${\mathrm{CD}}_{O_{2}}$ (ml/cm/s)	992 ± 197	680 ± 133^*^	−312 ± 125	797 ± 157^†^	577 ± 139^†*^	−222 ± 90^†^	**0.003**	**0.000**	**0.009**
Cardiopulmonary									
HR (bpm)	66 ± 15	69 ± 16	4 ± 7	62 ± 10	68 ± 12	6 ± 6	0.502	**0.000**	0.262
MAP (mmHg)	94 ± 15	87 ± 14	−8 ± 6	90 ± 24	84 ± 20	−6 ± 20	0.515	**0.004**	0.717
TPR (mmHg/l/min)	15.33 ± 4.21	13.08 ± 3.65	−2.25 ± 1.52	15.57 ± 3.92	12.33 ± 3.45	−3.24 ± 2.04	0.829	**0.000**	0.087
$P_{{\mathrm{ETCO}}_{2}}$ (mmHg)	41 ± 3	29 ± 5	−12 ± 5	39 ± 6	28 ± 5	−10 ± 8	0.210	**0.000**	0.451

Values are means ± SD. ^†^ *P* < 0.05 *vs*. controls. ^*^ *P* < 0.05 within groups. *P*‐values in bold indicate statistical significance. Abbreviations: ${\mathrm{CD}}_{O_{2}}$, cerebral oxygen delivery; CVCi, cerebrovascular conductance index; CVRi, cerebrovascular resistance index; DMCAv, diastolic middle cerebral artery blood velocity; HR, heart rate; MAP, mean arterial pressure; MCAv, middle cerebral artery blood velocity; $P_{{\mathrm{ETCO}}_{2}}$, end tidal carbon dioxide; PI, pulsatility index; SMCAv, systolic middle cerebral artery blood velocity; TPR, total peripheral resistance.

#### 3.5.2 Relative changes

**FIGURE 3:**
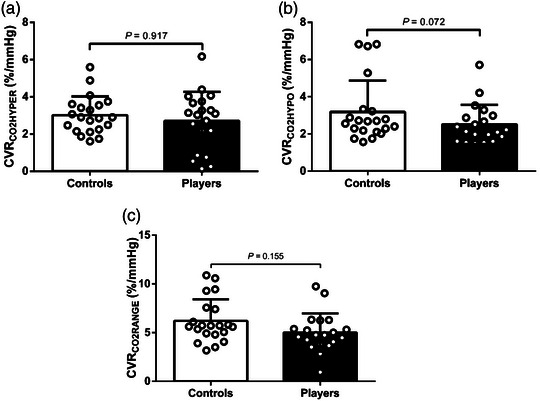
Cerebrovascular reactivity to hypercapnia (a), hypocapnia (b) and ${\mathrm{CVR}}_{{\mathrm{CO}}_{2}\mathrm{range}}$ (c). Values are means ± SD; controls, *n* = 21; players, *n* = 20. ${\mathrm{CVR}}_{{\mathrm{CO}}_{2}}$, cerebrovascular reactivity to changes in end‐tidal carbon dioxide.

In contrast, no differences were observed between groups for ${\mathrm{CVR}}_{{\mathrm{CO}}_{2}\mathrm{hyper}}$ (*U* = 206_(41)_, *P =* 0.917; Figure 3a), ${\mathrm{CVR}}_{{\mathrm{CO}}_{2}\mathrm{hypo}}$ (*U* = 141_(41)_, *P =* 0.072; Figure 3b) or ${\mathrm{CVR}}_{{\mathrm{CO}}_{2}\mathrm{range}}$ (*U* = 155_(41)_, *P =* 0.155; Figure 3c).

### 3.6 Cognition

MoCA scores indicated that 16 players were defined by MCI unlike controls (*P* = 0.020, 95% CI, −3.95 to −0.34; Table 6). Players also presented with a selective impairment in executive function and fine motor coordination of the non‐dominant hand (*U* = 141_(41)_, *P* = 0.021).

**TABLE 6:** Cognition.

Assessment	Controls (*n* = 21)	Players (*n* = 20)	*P*	95% CI
MCI screening				
Montreal cognitive assessment (*n*)	26 ± 2	24 ± 3	**0.020**	−3.95 to −0.36
Visuomotor coordination				
Grooved Pegboard dominant hand (s)	70 (12)	75 (15)	0.230	—
Grooved Pegboard non‐dominant hand (s)	78 (18)	86 (4)	**0.021**	—

Values are means ± SD or medians (interquartile range). P‐values in bold indicate statistical significance. MCI, mild cognitive impairment.

### 3.7 Correlations

We observed positive relationships between SCAT5 symptoms and symptom severity (*r* = 0.890, *P* < 0.001), bioactive NO and ${\mathrm{CVR}}_{{\mathrm{CO}}_{2}\mathrm{hyper}}$ (*r* = 0.567, *P* = 0.009), resting MCAv and ${\mathrm{CD}}_{O_{2}}$ (*r* = 0.905, *P* < 0.001), ${\mathrm{CVR}}_{{\mathrm{CO}}_{2}\mathrm{hyper}}$ and ${\mathrm{CVR}}_{{\mathrm{CO}}_{2}\mathrm{range}}$ (*r* = 0.798, *P* < 0.001). Negative relationships were observed between rugby career and ${\mathrm{CVR}}_{{\mathrm{CO}}_{2}\mathrm{hyper}}$ (*r* = −0.458, *P* = 0.042), SCAT5 symptoms and ${\mathrm{CVR}}_{{\mathrm{CO}}_{2}\mathrm{hypo}}$ (*r* = −0.480, *P* = 0.032), resting MCAv and ${\mathrm{CVR}}_{{\mathrm{CO}}_{2}\mathrm{hyper}}$ (*r* = −0.492, *P* = 0.028). No other correlations (*P* > 0.05) were observed for any molecular, cerebral haemodynamic or cognitive metrics (Table 7).

**TABLE 7:** Correlations.

	Age (years)	Rugby career (years)	Concussion history (*n*)	Concussion symptoms (*n*)	Concussion severity score (*n*)	NO_2_^−^ + RSNO (nM)	MCAv (cm/s)	${\mathrm{CD}}_{O_{2}}$ (ml/cm/s)	${\mathrm{CVR}}_{{\mathrm{CO}}_{2}\mathrm{hyper}}$ (%/mmHg)	${\mathrm{CVR}}_{{\mathrm{CO}}_{2}\mathrm{hypo}}$ (%/mmHg)	${\mathrm{CVR}}_{{\mathrm{CO}}_{2}\mathrm{range}}$ (%/mmHg)	MoCA (*n*)	GPND (s)
Age (years)													
*r*	1	0.205	−0.362	0.020	−0.125	−0.157	−0.060	−0.143	0.107	−0.053	0.066	0.093	0.322
*P*	–	0.387	0.117	0.935	0.599	0.508	0.800	0.547	0.653	0.823	0.783	0.696	0.167
Rugby career (years)													
*r*	0.205	1	0.210	0.108	0.156	−0.429	0.184	0.170	−0.458	0.196	−0.303	−0.414	0.184
*P*	0.387	—	0.373	0.650	0.512	0.059	0.438	0.474	**0.042**	0.408	0.194	0.069	0.436
Concussion history (*n*)													
*r*	−0.362	0.210	1	0.157	0.223	−0.231	−0.176	−0.163	−0.264	−0.100	−0.305	−0.260	−0.221
*P*	0.117	0.373	—	0.509	0.344	0.328	0.457	0.493	0.260	0.676	0.191	0.269	0.349
Concussion symptoms (*n*)													
*r*	0.020	0.108	0.157	1	0.890	0.092	−0.316	−0.259	0.053	−0.480	−0.246	−0.331	−0.442
*P*	0.935	0.650	0.509	—	**0.000**	0.701	0.175	0.269	0.823	**0.032**	0.295	0.154	0.051
Concussion severity score (*n*)													
*r*	−0.125	0.156	0.223	0.890	1	−0.010	−0.154	−0.084	−0.076	−0.310	−0.261	−0.421	−0.413
*P*	0.599	0.512	0.344	**0.000**	—	0.967	0.517	0.724	0.749	0.184	0.266	0.064	0.070
NO_2_^−^ + RSNO (nM)													
*r*	−0.157	−0.429	−0.231	0.092	−0.010	1	−0.188	−0.201	0.567	−0.197	0.403	0.300	−0.123
*P*	0.508	0.059	0.328	0.701	0.967	—	0.428	0.395	**0.009**	0.406	0.078	0.199	0.607
MCAv (cm/s)													
*r*	−0.060	0.184	−0.176	−0.316	−0.154	−0.188	1	0.905	−0.492	0.116	−0.383	0.105	0.010
*P*	0.800	0.438	0.457	0.175	0.517	0.428	—	**0.000**	**0.028**	0.626	0.095	0.659	0.968
${\mathrm{CD}}_{O_{2}}$ (ml/cm/s)													
*r*	−0.143	0.170	−0.163	−0.259	−0.084	−0.201	0.905	1	−0.336	0.191	−0.193	0.017	0.010
*P*	0.547	0.474	0.493	0.269	0.724	0.395	**0.000**	—	0.148	0.419	0.415	0.942	0.967
${\mathrm{CVR}}_{{\mathrm{CO}}_{2}\mathrm{hyper}}$ (%/mmHg)													
*r*	0.107	−0.458	−0.264	0.053	−0.076	0.567	−0.492	−0.336	1	−0.204	0.798	0.190	0.102
*P*	0.653	0.042	0.260	0.823	0.749	**0.009**	**0.028**	0.148	—	0.389	**0.000**	0.422	0.670
${\mathrm{CVR}}_{{\mathrm{CO}}_{2}\mathrm{hypo}}$ (%/mmHg)													
*r*	−0.053	0.196	−0.100	−0.480	−0.310	−0.197	0.116	0.191	−0.204	1	0.427	0.154	0.436
*P*	0.823	0.408	0.676	0.032	0.184	0.406	0.626	0.419	0.389		0.060	0.517	0.055
${\mathrm{CVR}}_{{\mathrm{CO}}_{2}\mathrm{range}}$ (%/mmHg)													
*r*	0.066	−0.303	−0.305	−0.246	−0.261	0.403	−0.383	−0.193	0.798	0.427	1	0.271	0.362
P	0.783	0.194	0.191	0.295	0.266	0.078	0.095	0.415	**0.000**	0.060		0.248	0.117
MoCA (*n*)													
*r*	0.093	−0.414	−0.260	−0.331	−0.421	0.300	0.105	0.017	0.190	0.154	0.271	1	0.075
P	0.696	0.069	0.269	0.154	0.064	0.199	0.659	0.942	0.422	0.517	0.248		0.754
GPND (s)													
*r*	0.322	0.184	−0.221	−0.442	−0.413	−0.123	0.010	0.010	0.102	0.436	0.362	0.075	1
P	0.167	0.436	0.349	0.051	0.070	0.607	0.968	0.967	0.670	0.055	0.117	0.754	

${\mathrm{CD}}_{O_{2}}$, cerebral oxygen delivery; ${\mathrm{CVR}}_{{\mathrm{CO}}_{2}\mathrm{hyper}}$ , cerebrovascular reactivity to hypercapnia; ${\mathrm{CVR}}_{{\mathrm{CO}}_{2}\mathrm{hypo}}$, cerebrovascular reactivity to hypocapnia; ${\mathrm{CVR}}_{{\mathrm{CO}}_{2}\mathrm{range}}$_,_ cerebrovascular reactivity range; GPND: Grooved Pegboard non‐dominant hand; MoCA, Montreal Cognitive Assessment; NO_2_^−^, nitrite; RSNO, *S*‐nitrosothiols; MCAv, middle cerebral artery blood velocity.

### 3.8 Symptom severity subgroups

We retrospectively divided players into high‐severity (*n* = 10) and low‐severity subgroups (*n* = 10), defined by those who scored above and below the median SCAT5 severity score (12 points). No between‐subgroup differences were observed in basal bioactive NO (*U* = 37.5_(21)_, *P* = 0.353, *r* = −0.253), MCAv (*P* = 0.253, 95% CI, −13.02 to 3.67, *r* = 0.268), ${\mathrm{CVR}}_{{\mathrm{CO}}_{2}\mathrm{range}}$ (*U* = 33.0_(21)_, *P* = 0.218, *r* = 0.261), MoCA (*P* = 0.347, 95% CI, −4.45 to 1.65, *r* = 0.222) and the GPND (*U* = 31.0_(21)_, *P* = 0.165, *r* = 0.286).

## 4 DISCUSSION

This study sought to define an integrated panel of molecular, haemodynamic and clinical biomarkers in retired rugby union players exposed to recurrent contact over an average playing career of 22 years with three previous concussions. Compared to matched controls, players were defined by lower systemic nitric oxide bioactivity and persistent neurological sequelae. This was accompanied by cognitive impairment as well as reduced MCAv both at rest and in response to hypercapnia/hypocapnia in the face of no molecular evidence of structural damage to the neurovascular unit (NVU). Collectively, these findings suggest that the accelerated cognitive decline caused by recurrent contact in rugby union may potentially be related to chronically impaired redox‐regulation of cerebrovascular function, highlighting the potential neuroprotective basis for targeted antioxidant prophylaxis and cognitive training.

### 4.1 Nutrient intake

There are no studies to the best of our knowledge that have documented nutrient intake in rugby union players and the potential link to altered OXNOS‐mediated cerebrovascular function. This is surprising given that inadequate intake of dietary antioxidants consumed in fruit and vegetables compounds OXNOS‐mediated vascular endothelial dysfunction and increases corresponding risk of cardiovascular disease and stroke (Wang et al., 2021). Furthemore, caloric restriction confers neuroprotective benefits by reducing obesity, insulin resistance and metabolic syndrome (Joseph et al., 2009). We chose to explore any potential differences in macro/micronutrient intake with a specific focus on those nutrients that could potentially impact systemic redox homeostasis given the intimate links observed with cerebrovascular function (Bailey, Culcasi et al., 2022; Fall et al., 2018).

It was intriguing to observe that players consumed fewer calories compared to their control counterparts and that dietary selenium intake was inadequate in all participants, particularly among players. The latter has been associated with cognitive decline and increased risk of Alzheimer's disease (Barchielli et al., 2022). Observational studies have identified an association between high homocysteine levels and increased incidence of dementia (Seshadri et al., 2002). Folate, which plays a key role in reducing homocysteine (Mann & Chisholm, 2017), was lower in players. Hence, it can be argued that an increased intake of folate, present in a wide variety of foods, including vegetables, especially dark green leafy vegetables and fruits, may be desirable in the players, in order to prevent the formation of the amino acid homocysteine. Therefore, long‐term dietary observations are recommended for retired athletes with concussion history and may offer valuable insights towards accelerated brain ageing.

In contrast, no between‐group differences were observed in the basal intake of ascorbate, the primary water‐soluble chain‐breaking antioxidant required for targeted ‘repair’ of superoxide, hydroxyl, alkyl, peroxyl, alkoxyl and tocopheroxyl radicals during chain propagation, the latter synergising regeneration of α‐tocopherol, the primary fat‐soluble chain‐breaking antioxidant (Sharma & Buettner, 1993) that was also comparable. Thus, we are confident, given the imposition/maintenance of dietary control, which is not commonplace in the published literature, that the observed differences in basal OXNOS biomarkers cannot simply be ascribed to dietary factors.

### 4.2 Molecular function

Our findings indicate that a markedly lower basal concentration of systemic NO, taking the form of lower NO_2_^−^ and RSNO, persists following retirement from rugby union. While the mechanism for reduced systemic NO bioactivity in the retired players remains to be determined, an accelerated downregulation of vascular endothelial nitric oxide synthase (eNOS) associated with natural ageing is plausible (Keske et al., 2016). Moreover, bioactive NO correlated positively with ${\mathrm{CVR}}_{{\mathrm{CO}}_{2}\mathrm{hyper}}$, confirming that the NO–CO_2_ axis is an important pathway in the chemoregulation of CBF (Lavi et al., 2003), although we cannot discount some degree of mechanoregulation given the observed (and anticipated) elevation in BP. Importantly, our experimental design and prospective focus on baseline matching excludes any contaminating contributions from differences in CRF, diet especially antioxidant vitamins, and medication, factors known to compound systemic OXNOS (Bailey et al., 2006; Bailey, Culcasi et al., 2022).

Owens et al. (2021) previously observed elevated systemic OXNOS in young professional players aged 25 (IQR, 5) years, whereby exposure to recurrent contact and concussion promoted mitochondrial dysfunction and elevated formation of free radicals that compete for/scavenge NO. While oxidative stress was not assessed in the present study, our previous findings suggest that retired players may have been exposed to prolonged elevated OXNOS throughout their 22 (IQR, 6)‐year playing careers that persists into retirement.

### 4.3 Neurovascular unit integrity/injury

There are no serum biomarkers capable of diagnosing or identifying the long term effects of concussion at present. However, authors of the ‘BRAIN Study’ have speculated that biomarkers related to axonal injury and neuroinflammation including NFL and total tau (t‐tau) are most likely to be detected in individuals with a longstanding history of head trauma (Gallo et al., 2017). We observed no differences in NFL, GFAP and NSE when comparing retired players with controls, seemingly due to higher variability between subjects and a smaller sample size compared to other reports. Therefore, our results suggest that these markers offer limited sensitivity and specificity when assessing long term damage following concussion in sport. However, such markers may have more clinical relevance when diagnosing and informing return to play decisions following concussion in sport (Marchi et al., 2013; Oris et al., 2022).

### 4.4 Haemodynamic function

Consistent with our original hypothesis, players were characterised by comparatively lower basal MCAv, ${\mathrm{CD}}_{O_{2}}$ and CO_2_ vasoreactivity. Therefore, recurrent contact and concussion history contributed to a decline in resting cerebral blood velocity and this concurs with previous findings in retired contact sport athletes (Hart et al., 2013). Given the brain has high metabolic turnover and is heavily reliant on sustained O_2_ and glucose delivery (Bailey, 2019), cerebral hypoperfusion has been associated with cognitive impairment and increased susceptibility to neurodegeneration (Bailey et al., 2019; Wolters et al., 2017). Likewise, cerebral hypoperfusion and persistent neurological sequelae have previously been observed (Meier et al., 2015), which correspond with the increased symptom severity observed in the players.

While we did not measure the extent of structural damage via neuroimaging, Grossman et al. (2013) have demonstrated that concussion promotes neuronal apoptosis via mechanically induced shear stress. Indeed, players were characterised by lower MCAv relative to controls, and it is important to consider that anterior hypoperfusion may prevail. Indeed, brain regions supplied via the middle cerebral artery have been characterised by hypoperfusion following concussion, namely the dorsal midinsular cortex, right superior temporal sulcus and regions of the frontal lobe (Churchill et al., 2017; Meier et al., 2015; Uddin et al., 2017). Under these circumstances, areas of increased vulnerability may arise due to O_2_ and glucose delivery that is favoured towards vascular beds that are not compromised by regional hypoperfusion following injury (Ellis et al., 2016; Grossman et al., 2013).

In an apparently healthy population, the age‐related decline in resting MCAv is 25–30% (Ainslie et al., 2008). In support, we are able to demonstrate an accelerated decline in MCAv across the lifespan when comparing the results of the present study with those of our previously published work in young professional rugby union players aged 25 (IQR, 5) years (Owens et al., 2021). We observed a comparable (26%) reduction in MCAv when comparing the young controls of our previous study (Owens et al., 2021) and the aged controls in the present study. When comparing the young control group and the retired players, MCAv had declined by 41%. Despite the accelerated decline in basal MCAv in retired players, all remained within normative ranges (34–86 cm/s, Adams et al., 1992). Therefore, interpretative caution must be applied when considering the clinical significance of these findings.

In contrast to our original expectations, we failed to observe any between‐group differences in ${\mathrm{CVR}}_{{\mathrm{CO}}_{2}}$ (Bailey, Jones et al., 2013). Brugniaux et al. (2014) have previously demonstrated the importance of challenging the cerebrovasculature to reveal subtle impairments across the human ageing continuum. In response to ${\mathrm{CVR}}_{{\mathrm{CO}}_{2}\mathrm{hyper}}$ and ${\mathrm{CVR}}_{{\mathrm{CO}}_{2}\mathrm{hypo}}$, no differences were identified between players and controls. However, ${\mathrm{CVR}}_{{\mathrm{CO}}_{2}\mathrm{hyper}}$ was (negatively) correlated with the number of playing years, highlighting the impact a prolonged career in professional rugby may have on the cerebrovasculature. Furthermore, when absolute changes in MCAv and ${\mathrm{CD}}_{O_{2}}$ were observed between groups independent of consequent differences in $P_{{\mathrm{ETCO}}_{2}}$, selective reductions were observed among players. According to our approach, the assessment of cerebrovascular function appears a more sensitive ‘dynamic’ biomarker of concussion history in retired athletes compared to molecular blood‐borne biomarkers, given that impairments in cerebral haemodynamic function were present in the absence of damage to the NVU and particularly the BBB. Given that players engaged in more physical activity than the UK guidelines for both aerobic and resistance exercise (Public Health England, 2020), the impairment in cerebral haemodynamic function is likely underestimated and would likely be further compromised in the (more) sedentary demographic.

### 4.5 Cognitive function

Sixteen players presented with MoCA scores that were indicative of MCI, complimented by lower executive function and fine‐motor coordination of the non‐dominant hand. Collectively, prior recurrent contact and concussion history ‘accelerates’ the decline in cognition observed during ‘normal’ sedentary ageing. These impairments were independent of physical inactivity‐induced degeneration, given that both groups were matched for CRF and again likely underestimated in the (more) sedentary demographic. Neurodegenerative diseases are often preceded by MCI, with 10–20% of MCI patients transitioning towards neurodegeneration annually in comparison to 1–2% of non‐MCI patients (Meyer et al., 2002; Tierney et al., 1996). While structural damage, decreased nitric oxide bioactivity and suppressed cerebral haemodynamic function are likely contributors, the mechanisms are not fully understood and require further research (McMillan et al., 2017).

While concussion incidence between the ‘amateur’ and professional eras of rugby are largely different (Gardner, Iverson, Williams et al., 2014; Owens et al., 2019), tackles remain the primary cause of concussion, particularly when involving head‐to‐head collisions (Cross et al., 2017; Owens et al., 2019). Moreover, these match events increase the risk of damage to both the frontal and temporal lobes given that contact is most frequent in the anterolateral regions of the head during play (Pearce et al., 2018; Tarazi et al., 2018). Neuroimaging studies of concussed athletes have demonstrated structural and functional deficits in these brain regions (Churchill et al., 2020; Grossman et al., 2013; Meier et al., 2015). Thus, it is not surprising that we observed MCI and impaired fine‐motor coordination of the non‐dominant hand in players, given that these cognitive domains are governed by the dorsolateral prefrontal cortex, anterior cingulate cortex and orbitofrontal cortex of the frontal lobe (Miller & Cohen, 2001).

### 4.6 Limitations

There are a number of study limitations that warrant careful consideration. We were not able to assess global free radical formation using direct biomarkers including the electron paramagnetic resonance spectroscopic detection of the ascorbate free radical (A^•−^) as the ‘upstream’ catalyst of OXNOS that we have previously documented in young players (Owens et al., 2021). Larger scale follow‐up studies using biomarkers of NVU integrity are further encouraged to confirm our findings given the interpretive limitations associated with the small sample sizes employed, including the caveats associated with a Type M error (Gelman and Carlin, 2014). Furthermore, an attempt to record the number of contact events across the playing career of players would have been equally insightful, but was not possible given the varied injury reporting methods documented in the amateur era of the game when participants of this research study would have been competing (Owens et al., 2019). Similarly, it was not possible to collect additional information pertaining to concussion severity. While we retrospectively classified players into high‐severity and low‐severity subgroups based on SCAT5 severity scores, no differences were observed, likely due in part to inadequate statistical power.

The FFQ implemented in this study assessed typical food intake over a 12‐month period. Therefore, considerations for acute dietary differences were not possible. Future investigations should therefore seek to better understand the relationship between OXNOS, NVU integrity (dynamic contrast‐enhanced and dynamic susceptibility contrast MRI neuroimaging), cerebral haemodynamic and cognitive function in retired contact sport athletes. Similarly, better reporting of match events should be considered, particularly at the amateur levels of play given the potential consequences of recurrent contact on player welfare.

### 4.7 Conclusion

Retired rugby union players with a history of multiple concussions and two‐decade long playing careers at regional and international level presented with impairments in molecular, cerebral haemodynamic and cognitive function compared to non‐concussed, non‐contact controls. While this study observed rugby players, the findings of this research may translate to a wider range of ‘contact’ sports characterized by recurrent concussion, including albeit not exclusively confined to, boxing, mixed martial arts, soccer and American football, hockey and horse‐racing. Targeted interventions that minimise the risk of accelerated cognitive impairment following concussion are warranted. Given that molecular and cerebral haemodynamic profiling may serve as useful biomarkers to manage player welfare and predict longer term clinical trajectory, longitudinal observations should be considered in current players.

## AUTHOR CONTRIBUTIONS

Obtained funding: Damian M. Bailey Conception and design of work: Thomas S. Owens, Christopher J. Marley, Gareth L. Jones, Damian M. Bailey Acquisition of data for the work: Thomas S. Owens, Christopher J. Marley, Thomas A. Calverley, Benjamin S. Stacey, Lewis Fall, Hayato Tsukamoto, Angelo Iannetelli, Teresa Filipponi, Bruce Davies, Gareth L. Jones, Christophe Hirtz, Sylvain Lehmann, Edouard Tuaillon, Nicola Marchi, Damian M. Bailey Analysis of data for the work: Thomas S. Owens, Gareth L. Jones, Christophe Hirtz, Sylvain Lehmann, Edouard Tuaillon, Nicola Marchi Interpretation of data for the work: Thomas S. Owens, Gareth L. Jones, Damian M. Bailey Drafted the work: Thomas S. Owens, Christopher J. Marley, Teresa Filipponi, Gareth L. Jones, Damian M. Bailey Revised the work for important intellectual content: Thomas S. Owens, Christopher J. Marley, Thomas A. Calverley, Benjamin S. Stacey, Lewis Fall, Hayato Tsukamoto, Angelo Iannetelli, Teresa Filipponi, Bruce Davies, Gareth L. Jones, Christophe Hirtz, Sylvain Lehmann, Edouard Tuaillon, Nicola Marchi, Damian M. Bailey All authors approved the final version of the manuscript and agree to be accountable for all aspects of the work in ensuring that questions related to the accuracy or integrity of any part of the work are appropriately investigated and resolved. All persons designated as authors qualify for authorship, and all those who qualify for authorship are listed.

## CONFLICT OF INTEREST

D.M.B. is Editor‐in‐Chief of *Experimental Physiology*; a member of the ‘Concussion in Sport Committee’ for The Department for Digital, Culture, Media & Sport (c/o Medical Research Council); Chair of the Life Sciences Working Group; member of the Human Spaceflight and Exploration Science Advisory Committee to the European Space Agency and member of the Space Exploration Advisory Committee to the UK Space Agency; and affiliated to the companies FloTBI Inc. and Bexorg Inc. focused on the technological development of novel biomarkers of brain injury in humans.

## Data Availability

The datasets used and/or analysed during the current study are available from the corresponding author on reasonable request.
